# Cardiac fibrosis and down regulation of GLUT4 in experimental diabetic cardiomyopathy are ameliorated by chronic exposures to intermittent altitude

**DOI:** 10.15171/jcvtr.2016.05

**Published:** 2016-03-14

**Authors:** Mahdi Faramoushi, Ramin Amir Sasan, Vahid Sari Sarraf, Pouran Karimi

**Affiliations:** ^1^Department of Physical Education and Sport, Tabriz Islamic Art University, Tabriz, Iran; ^2^Faculty of Physical Education and Sport Sciences, University of Tabriz, Tabriz, Iran; ^3^Neuroscience Research Center (NSRC), Tabriz University of Medical Sciences, Tabriz, Iran

**Keywords:** Cardiac, Fibrosis, GLUT4, Cardiomyopathy, Altitude

## Abstract

***Introduction:*** Chronic intermittent hypoxia is considered as a preconditioning status in cardiovascular health to inducing resistance to the low oxygen supply. Diabetic cardiomyopathy leads to inability of the heart to effective circulation of blood preventing of consequent tissue damages so; the aim of this study was elucidation of effect of chronic exposure to hypoxia on Cardiac fibrosis and expression of GLUT4 in experimental diabetic cardiomyopathy.

***Methods:*** A total number of 30 rats were randomly divided into three groups; 1: Normoxia control group (NN, n = 10). 2: Normoxia diabetic group (ND, n = 10) that took fat diet for 2 weeks then were injected by streptozotocin (37 mg/kg) and 3: Hypoxia diabetic group (HD, n = 10): that were exposed to chronic intermittent hypoxia (CIH) (altitude ≈3400 m, 14% oxygen for 8 weeks). After hypoxia challenge, plasma metabolic parameters including: fasting blood glucose (FBS), triglyceride (TG) and total cholesterol (TC) were measured by colorimetric assay. Cardiac expression of GLUT4 protein and cardiac collagen accumulation were determined in the excised left ventricle by western blotting, and Masson trichrome staining respectively.

***Results:*** Based on resultant data, FBS, TG and TC were significantly (*P* < 0.05) decreased in HD vs. ND. Homeostasis Model Assessment (HOMA) were also significantly attenuated after exposed to CIH in HD group compared to ND group (*P* < 0.05). Significant increase in packed cell volume and hemoglobin concentration was observed in HD group compared to ND group (P < 0.05). Comparison of heart wet weight between three groups showed a significant difference (*P* < 0.05) with lower amount in HD and ND versus NN. Myocardial fibrosis was significantly more pronounced in ND when compared to NN. Eight weeks exposure to hypoxia ameliorated this increase in HD group. Intermittent hypoxia significantly increased GLUT4 protein expression in HD compared to ND group (*P* < 0.05).

***Conclusion:*** Data suggested that CIH might potentiate to improve glucose homeostasis and cardiac tissue structural damages created in type 2 diabetes (T2D).

## Introduction

Type 2 diabetes (T2D) is a chronic metabolic disorder considered to be the fifth cause of death in the world after infectious diseases, cardiovascular diseases, cancer and trauma is accompanied with insulin resistance of the whole body and myocardial cells.^1^ This type of diabetes is the most common type of mellitus diabetes and is a serious threat for the health of mankind in the 21st century and due to its rapid expansion, has attracted much attention in the last two decades.^2^ Type 2 diabetic people are threaten by cardiovascular diseases and cardiac failure. About 30 years ago, Rubler et al showed cardiac insufficiency in diabetic patients who had normal coronary artery and blood pressure. This kind of cardiac disease which is caused by diabetes and is independent on weight, levels of cholesterol and age is called cardiomyopathy.^3^ In cardiomyopathy, the heart extensively suffers from fibrosis, apoptosis, and abnormal hypertrophy, which can be resulted from irregularity in cardiac metabolism related to glucose homeostasis. In glucose deprivation condition, the normal dependency of the heart to fat metabolism for energy supply increases which is commonly accompanied by decreasing in the glucose trafficking across plasma membrane of myocardium cells by glucose transporters (GLUTs).^1,4^

GLUT4 is the most important transferring isoform of glucose, which is extensively expressed in insulin sensitive cells such as adipose tissues, skeletal muscles, and cardiomyocytes. Any kind of down regulation or conformational change in it cause to its malfunction, which is commonly happens in diabetes, leads to irregularity in glucose homeostasis. The capacity of GLUT4 synthesis also fall down inside the cells^5,6^ in T2D.

On the other hand, unknown hypertrophy in diabetic patients is without noticeable symptoms, which, according to the recent findings, is experienced by 56% of the diabetic patients. Myocyte hypertrophy is common in the biopsy of the diabetic heart.^7^ In some diabetic model rats, increased cardiac mass has been reported in 9 and 12 weeks. Moreover, an increase in the left ventricular (LV) mass and its septum thickness in streptozotocin-induced diabetic has been also reported.^8^

T2D is identified by the utilization decrease of glucose, the consumption increase of fatty acid, calcium-handling disorder, compromised mitochondrial energies, and the increase of cardiac connective tissues^9^ which are leading to induction of apoptosis by triggering of Fas receptors.^10^

Altitude is a potential conditions for the reduction of morbidity and mortality rates for 400 million people who live around 1500 m above the sea level.^11^ The findings from the population of Andes indicate that the incidence of heart diseases low among people who live in high altitudes.^12^ In a research done in altitude of around 4260 m, no instances of cardiac infraction or coronary heart disease are found.^13^ It seems that the symptoms of moderate altitude appear quickly as a moderate altitude like stress may result in the reconstruction of‏ cardiorespiratory reflections.^12^

Voors and Johnson have found a negative relationship between altitude and mortality in several big cities with more than 1650 m above sea level in the United States.^14^ This finding has also been confirmed with other recent studies.^13,15^ In Switzerland, the age mortality rate caused by cardiovascular diseases among 100 thousand people living in an altitude of above 1500 m has been shown to have a meaningful decrease among both men and women.^16^ High altitude may also have a protective role against cardiovascular and chronic respiratory diseases.^17^

Residence in a condition of hypoxia especially its intermittent type mimics a conditions like live in altitude creates. Chronic intermittent hypoxia (CIH) is considered as a useful experimental condition in cardiovascular health. From biological point of view, it is supposed that HIF-1 (Hypoxia Inducible Factor-1) may play a central role in pathophysiology of hypoxia.^18^

Moreover, it has already been shown that if rats be placed in hypoxic condition, their cardiac structure will changed. It is also reported that glucose consumption by heart is increased (by 70%) in simulated altitude hypoxia (14%-15% of oxygen saturation). Regular and chronic exercise in hypoxic condition can increase glycogen supply and glucose tolerance as well as raised expression of GLUT4.^19^ Similar finding was shown in Dill et al study on prolonged hypoxia which could induce GLUT4 expression and improve glucose metabolism in the cardiomyocytes and the whole body cells.^20^

Despite clear evidence in effect of altitude, only a few studies are available on the mechanism of effect of altitude on mortality rate caused by common diseases.

Also, the effect of altitude on type 2 diabetic patients, especially on their heart, has been considered even much less.

Therefore, further research in this field can shed light on mechanism of effect of altitude or CIH. Therefore, our hypothesis is that CIH may affect cardiac structure and metabolism that subsequently, may lead to improve diabetic cardiomyopathy. So, in this study, we aimed to know whether cardiac fibrosis, hypertrophy and down regulation of GLUT4 are ameliorated by chronic exposures to intermittent altitude in experimental diabetic cardiomyopathy.

## Materials and methods

### Animals

30 Male Wistar rats weighed 200-220 g at the start of experiment. Rats were bought from Pasteur Institute (Tehran, Iran) and were acclimatized to our lab for a period of one week before starting the intervention. Each rat was housed alone in one regular cage and was maintained less than 12 hours light/dark cycles in a quiet environment with 50% humidity and 22±1°C temperature. The animals were fed standard lab rat chow dieting water ad libitum. Animals were equally divided into three groups: Normal normoxia group (NN, n=10): healthy control rats living in normoxic conditions. Normoxia diabetic group (ND, n=10): Diabetic control rats receiving fat rich diet for 2 weeks before intraperitonealy receiving streptozotocin (Sigma Aldrich, USA) also living in normoxic conditions, and hypoxia diabetes group (HD, n=10): rats treated in similar conditions with ND group but were exposed to CIH.

### Induction of diabetes

Before injection, rats were fed with high-fat diet including carbohydrates (35%), proteins containing all essential amino acids (15%), lipids (50%), vitamins (0.5%) and minerals for 2 weeks. Then rats were assigned to the diabetic groups and injected intra-peritoneal (i.p.) with 37 mg/kg streptozotocin citrate buffer (0.1M, pH 4.5).^21^ 3-4 days after the injection of STZ, to confirm the induction of diabetes fasting blood glucose (FBG) measured in one-drop blood sample obtained from vein tail. FBG levels were rapidly measured by a digital blood glucose meter (Accu-Chek Sensor, Roche, Mannheim, Germany). FBG more than 300 mg/dL were confirmed as diabetic rats and were included into the study.

### Simulation altitude

Chronic exposure to intermittent hypoxia (CIH) was performed by an environmental hypoxic chamber while rats were kept on it for 8-12 h/day and accessed to food and water and libitum. Hypoxia was made by placing animals (HD group) in a one large room chamber (length=80 cm; width=50 cm; high=150 cm) with a partial pressure of 14% for oxygen. A balanced flow of the partial pressure of oxygen of 14% (PIO2 ≈106 mm Hg, altitude ≈3400 m) was created inside the chamber all over the 12-hour period, and an alarm oxygen sensor was used to check the oxygen concentration. (GO2Altitude, Biomed tech, Australia Pty. Ltd, Melbourne).^22^ Normoxia groups including normal and diabetic groups were maintained for 8 weeks at baseline (Tabriz, Iran) (PIO2 ≈159 mm Hg, altitude ≈1100 m).

### Sample collection

In the end of experiment, after 12 hours fasting, rats were deeply anesthetized with ketamine HCl (100 mg/kg) and xylazine (10 mg/kg) i.p. By using a syringe, Blood samples were totally withdrawn from the heart and divided into two tubes, one containing ethylenediaminetetraacetic acid (EDTA) and another one as serum separator tube without any anticoagulant to prepare plasma and serum, respectively. In serum tube, supernatant was collected by centrifuge at 3000 × g for 15 minutes after clot formation at RT. All serum samples were liquated and kept at −20°C to avoid repeated freeze–thaw. Whole blood was subjected to measurement of hematologic parameters by cell counter (Exigo-vet, Sweden). The heart of each animal was cutout from the chest and after removing of atria and large vessels was weighed. A mid LV section was vertically cut and fixed in 10% formaldehyde. Some ventricles tissues were quickly frozen in liquid nitrogen and were kept at -80°C for following immunobloting assay.

### Biochemical measurements

Glycemic parameters including FBG and fasting blood insulin (FBI) were measured by enzymatic end procedure and enzyme linked immunosorbent assay respectively**.** FBG, total cholesterol (TC) and triglyceride (TG) determined in serum using Pars Azmoon kits (Tehran, Iran). Homeostasis model of insulin resistance (HOMA-IR) was calculated as described:

HOMA–IR (Mass units) = fasting insulin (mIU/ml) × fasting glucose (mg/dl)/405 as well as quantitative insulin sensitivity check index (QUICKI) was calculated by calculating the inverse of the sum of logarithmically expressed values of FBG and fasting insulin.^23^

### Enzyme linked immunosorbent assay

The levels of plasma fasting insulin were determined by quantitative Sandwich ELISA kit (Cat No; 90010). Briefly, monoclonal antibody-coated microliter plate wells were loaded by 50 µl standards or samples. In order to determine quantitative amount of insulin in the sample, a horseradish peroxidase (HRP)-conjugated polyclonal antibody, specific for rat insulin was added to each well. The wells underwent three times washing with PBS 1x following incubations 1 hour at RT. Next, TMB substrate as chromogenic solution was added and allowed to react over a 20 minutes. The chromogenic reaction was halted by addition of stop solution, then absorbance of wells was read using spectrophotometer (awareness, USA) at 450 nm. The amount of insulin concentration in each sample was determined by providing standard curve (X=concentrations and Y=OD).

### Histological examination

The left ventricle myocardial muscle, (n=4 from each group) was fixed in 10% formaldehyde. Paraffin embedded sections were subjected to stain with hematoxylin and eosin (H&E) or Masson trichrome. Three sections with 10 fields per each section for each sample, (n=30 per rat) were analyzed. After scanning and computation with an image analyzer software (WinRoof, Mitani Co), the mean of fibrotic elements rate particular collagen, was calculated in the images. Analysis of variance (ANOVA) was used to compare of means.

### Western blotting

The samples of cardiac tissues were homogenized in ice-cold RIPA lysis buffer (50 mMTris-HCl, pH 8.0, 0.1% sodium dodecyl sulfate, 150 mM sodium chloride, 0.5% sodium deoxycholate and 1.0% NP-40). The supernatant was separated from debris by centrifugation at 12000 × g for 15 minutes at 4°C. The Bradford assay was applied to determine protein concentration (Bio Rad). A mixture 1:1 (v/v) of sample with 2X loading buffer was made and then boiled for 5 minutes. Fifty micrograms protein from each sample was subsequently loaded onto a 10% denaturing polyacrylamide mini gel. The protein bands were transferred to a methanol-preactivated polyvinylidene fluoride (PVDF) membrane. The membrane was blocked within bovine serum albumin (BSA) 1% in phosphate-buffered saline (PBS) plus 0.1% tween 20 for 2 hours with gently shake. Subsequently, the membrane was incubated overnight at 4°C with rabbit anti- GLUT4 (sc-7938; diluted 1:300 Santa Cruz Biotechnology Inc, Santa Cruz, CA, USA). The membrane was underwent three times washing with PBST and incubated for 1hour with horseradish peroxidase-conjugated goat anti rabbit antibody (sc-2030; diluted 1:6000 Santa Cruz Biotechnology Inc., Santa Cruz, CA, USA). Immune complex was visualized using enhanced chemiluminescence. Density of GLUT4 and β-actin references bands were quantified using Image J software, and relative density of each target protein signal was calculated to β-Actin.

### Statistical analysis

The results were expressed as mean ± SEM and analyzed using SPSS (version 18). To compare of means we used of ANOVA followed by Tukey test as post hoc test. *P*<0.05 was considered as statistical significance.

## Results

### Mortality and survival

There was no mortality in control group throughout experiment. But we lost four animals in each of ND (2/10) and HD (2/10) groups.

### Metabolic indexes

FBI was significantly decreased in STZ-diabetics groups compared to normoxia normal group (*P*<0.05). Table 1 shows CIH caused a diminish in FBG concentration after 8 weeks (243.250±24.5229; *P*=0.02) compared to ND group. HOMA-IR was significantly improved after exposure to hypoxia in HD group (2.14±0.18) compared to ND group (3.4400±0.2441) (*P*<0.05). In addition, total serum cholesterol and TG levels were significantly decreased in HD compared to ND group (*P*<0.05; Table 1).

**Table 1:** Effect of hypoxia on serum levels of metabolic parameters and biomarkers of diabetic’s rat

	**Normoxia control (n=10)**	**Normoxia diabetes (n=8)**	**Hypoxia diabetes (n=8)**
Fasting blood glucose (mg/dl)	104.68 ± 5.01	356.53 ± 40.15^a^	243.25 ± 24.52^b^
Fasting blood insulin (µU/L)	5.10 ± 0.14	3.44 ± 0.24^a^	3.68 ± 0.31
QUICKI	1.18 ± 0.01	0.92 ± 0.03^a^	0.97 ± 0.04
HOMA-IR	1.31 ± 0.08	3.52 ± 0.38^a^	2.14 ± 0.18^b^
Total serum cholesterol (mg/dL)	67.12 ± 1.31	76.00 ± 1.79^a^	68.66 ± 2.10^b^
TG (mg/dL)	50.87 ± 1.97	57.37 ± 2.66^a^	49.16 ± 1.77^b^

Abbreviation: FBG‏, fasting blood glucose; FBI, fasting blood insulin; TG, triglyceride; HOMA-IR, homeostasis model assessment (HOMA) of insulin resistance; QUICKI, Quantitative insulin sensitivity check index. ^a^*P*<0.05 compared with control group. ^b^*P*<0.05 compared with Diabetes group. Data are represented as mean ± SEM.

### Hematological parameters

The hematologic data were showed in Table 2. Significant decrease in red blood cell (RBC), hemoglobin (Hb) and hematocrit percentage (HCT%) were observed in ND group compared to NN group. Whereas significant increase in HCT%, Hb concentration and mean corpuscular hemoglobin (MCH) were observed in HD group compared to ND group (*P<*0.05). However, the total white blood cell (WBC) and lymphocyte counts were increased in the ND group but hypoxia return them to normal levels. WBC count was not significantly changed in the diabetic groups (Table 2).

**Table 2:** Effect of hypoxia on serum levels of hematologic parameters of diabetic rats

	**Normoxia control (n = 10)**	**Normoxia diabetes(n = 8)**	**Hypoxia diabetes (n = 8)**
WBC (10^9^/L)	3.25±0.20	6.12±0.82^a^	3.78±0.48^b^
LYM (10^9^/L)	1.80±0.13	3.75±0.59^a^	1.76±0.49^b^
MON (10^9^/L)	0.21±0.04	0.31±.04	0.25±0.05
Gran (10^9^/L)	1.24 ±0.07	2.06 ±0.33	1.76± 0.28
HCT (%)	38.34±0.77	33.51±0.61^a^	40.83±0.83^b^
Hb (g/dl)	12.78±0.25	11.17±0.20^a^	13.61±0.27^b^
MCH (pg)	18.31±0.73	18.54±1.03	25.06±0.85^ab^
RBC (10^12^/L)	7.03± 0.24	6.11± 0.25^a^	5.46± 0.20^a^

Abbreviation‏: WBC; white blood cell, LYM; lymphocyte, MON; monocyte, Gran; granulocyte, HCT; hematocrit, Hb; hemoglobin, RBC; red blood Cell, MCH; mean corpuscular hemoglobin, ^a^*P* < 0.05 compared with control group. ^b^*P* < 0.05 compared with diabetes group. Data are represented as mean ± SEM.

### Body weight and hypertrophic parameters

The initial mean body weights of the rats in the three groups before treatment did not differ. At the end of the 8-week CIH, the body weight of the ND group was significantly lower than that of the NN group (*P<*0.05) (Table 3). At the end of the study period, the means of heart wet weight among the three groups showed a significant difference (*P<*0.05; Table 3) with a lower weight in diabetic groups (ND and HD). Eight weeks treatment in hypoxic condition, could not improve weight loss in heart. There was no significant difference in the heart to body weight ratio between diabetic animals compared to normoxia normal control rats (3.5792±0.13633 vs. 3.8457±0.14081) as well as HD did not showed any change in this ratio. The ventricular weight was significantly decreased in ND group (0.4438±0.01861) but there was no significant difference in the means of LV weight between NN group (0.5763±0.02834) and HD group (0.5117±0.03380). The hypoxia treatment significantly increased the LV to heart weight ratio, compared to normoxia diabetic group and backed to the normal state in NN group without altering the heart to body weight ratio (Table 3).

**Table 3:** Heart weights and body weights parameters. Data are represented as mean ± SEM

	Normoxia control (n=10)	Normoxia diabetes (n=8)	Hypoxia diabetes (n=8)
Initial BW (g)	245.75 ± 6.04	246.00 ± 6.84	237.16 ± 5.75
Final BW (g)	298.12 ± 3.49	274.37 ± 10.07^a^	269.83 ± 9.50
Heart wet wt (g)	1.14 ± 0.04	0.97 ± 0.03^a^	0.97 ± 0.04
LV wet wt (g)	0.57 ± 0.02	0.44 ± 0.01^a^	0.51 ± 0.033
Heart wt/body wt × 1000	3.84 ± 0.14	3.57 ± 0.13	3.63 ± 0.14
LV wt/heart wt × 10	5.02 ± 0.14	4.54 ± 0.08	5.23 ± 0.26^b^

Abbreviations: wt; weight, LV; left ventricular. ^a^ *P* < 0.05 compared with control group. ^b^P < 0.05 compared with diabetes group.

### Histopathological examination

**Figure 1:**
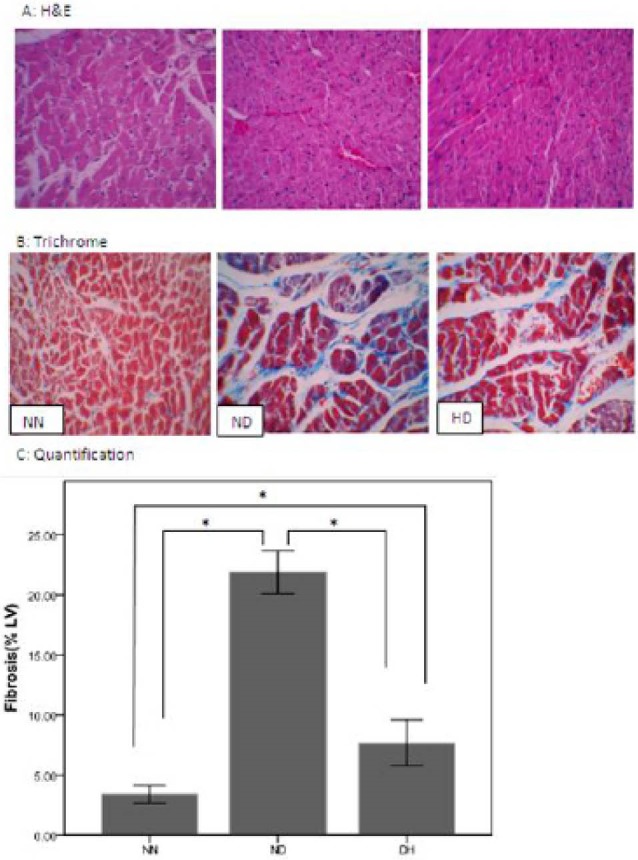
Histopathological analysis of left ventricular (LV) tissue sections stained with (A) Hematoxylin and Eosin (B) Masson trichrome and (C) Quantification of interstitial fibrosis with Masson trichrome stain were measured after 8 week from normoxia normal control group (NN), normoxia diabetes (ND), and chronic intermittent hypoxia diabetes (HD). Where blue = fibrous collagen, and red = cardiomyocytes 400X magnification. *P < 0.05.

The ventricular myocardium under normoxia showed normal architecture (Figure 1B and C). The characteristics of diabetic cardiomyopathy including disarray and myocardial degeneration were found in LV sections of the diabetic groups with hematoxylin-eosin staining (Figure 1A). The ventricular myocardium under normobaric hypoxia showed less disarrangement and abnormality in architecture vs. normoxia diabetic group. Myocardial fibrotic remodeling, as reflected by trichrome staining of myocardial sections, was significantly more pronounced in the ND, when compared to NN and HD groups (*P*<0.05). This increase in HD group was lower than ND group (Figure 1B). Semi-quantitative scoring of the staining showed a significant difference in LV fibrosis between the three groups (Figure 1C).

### GLUT4 expression

**Figure 2:**
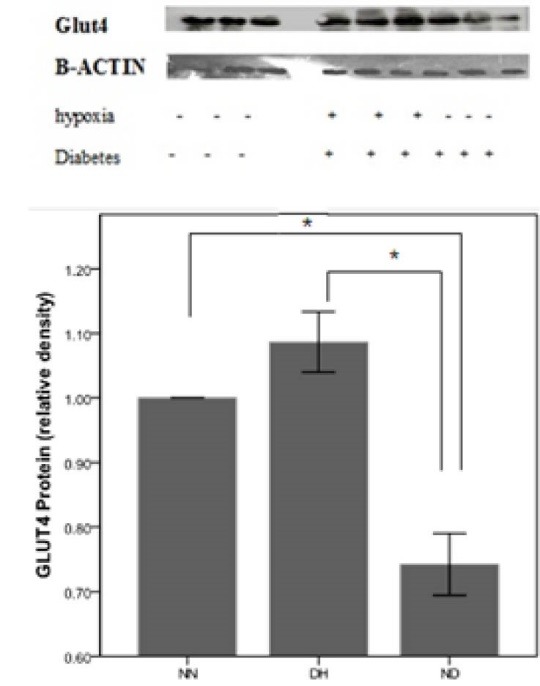
Effects of chronic and intermittent hypoxia on GLUT4 expression in diabetic rats. Extracts of left ventricle tissues from normoxia normal control (NN), normoxia diabetic control (ND) and hypoxic diabetic (HD) rats were subjected to Western blots. Expression of Glut4 in cardiac tissue (upper) and quantitation of immunoblotting (below), presented as the ratio to NN group. (Expression level of NN group was normalized as 1, n=3). *P< 0.05.

The Heart GLUT4 protein expression was measured 24 hours after exposure to hypoxia. The resultant data from immunoblotting assay of GLUT4 protein in ND and HD were presented as the relative density of NN group. STZ induced diabetes significantly decreased GLUT4 Protein expression compared to NN group (Figure 2), but hypoxia significantly increased GLUT4 protein expression in heart compared with ND group (*P*<0.05).

## Discussion

To the best of our knowledge, this is the first time that a study evaluates effect of CIH on diabetic cardiomyopathy. In the current study, the most striking findings are that increased expression of GLUT4 protein decreased collagen accumulation in cardiac tissue and improved glucose homeostasis in CIH diabetic rats vs. diabetic rats. Increased GLUT4 immunoreactivity was in compliance to Chou et al findings; They observed a significant increase in GLUT4 protein under 4 weeks hypoxia in rats cardiac muscle.^24^ Increased expression of GLUT4 protein in the heart may suggest elevated expression of GLUT4 in all muscles of the body.^10^ Another important finding of this study is that CIH as a simulation of altitude suppressed diabetes-induced hyperglycemia and elevated HOMA-IR that partially resulted from increased GLUT4 protein expression in the heart tissue cells. Furthermore, chronic and intermittent hypoxia treatment could rearrange lipidic parameters such as plasma levels of TC and TG. Similar to our work, Mackenzie et al showed that intermittent hypoxia caused to increased acute glucose clearance in T2D.^23^

Energy deprivation associated with hypoxia and pressure overload leads to the activation of AMP-activated protein kinase (AMPK) in the myocytes.^25^

AMPK, a serine/threonine protein kinase, acts as a fuel sensor responsible for mediating the cellular adaptation to nutritional and environmental stress.^26^ AMPK has important metabolic roles in cardiac muscle.^25^ Activation of AMPK by 5-aminoimidazole-4-carboxamide-1-β-D-ribofuranoside (AICAR) increases muscle glucose uptake in vivo and in vitro by a phosphatidyl-inositol 3-kinase independent mechanism. AMPK has also been implicated to have a key role in the stimulation of glucose transport in the ischemic heart.^27^

Activation of AMPK leads to translocation of GLUT4 glucose transporters to the cell surface.^26^ However, the events downstream from AMPK that modulate GLUT4 translocation are largely unknown.^28^ Modified regulation of cardiac metabolism in T2D has been well established. In diabetic states, myocardial O_2_ consumption and the normal dependency of fat metabolism are increased and cardiac function is decreased. In type 2 diabetic patients a 2-fold increase in heart palmitate oxidation and a 30%-40% decrease in glucose oxidation have been shown, and finally leading to cardiomyopathy in diabetic hearts.^29^

For a long time, it was thought that the Randle cycle was the main contributor to the biochemical shift towards fatty acid (FA) uptake and oxidation in T2D. Randle proposed that a higher rate of release of FAs and ketone bodies for oxidation was responsible for the decrease in GLUT4 protein levels.^30^ Therefore, increased expression of GLUT4 in CIH can rely on decreased fat metabolism in the heart and possible improved cardiomyopathy. Diabetic cardiomyopathy includes structural abnormality and functional defect of the myocardium lacking coronary artery disease.^31-33^ The hallmark histopathologic features of diabetic cardiomyopathy are interstitial and perivascular fibrosis.^34^ Based on our finding collagen deposition diminished in chronic hypoxic condition. In addition to influence of enhanced collagen deposition on cardiac failure, cross-linking of collagen fibers may be increased impaired glucose metabolism in diabetes.^34^ This result was similar to our result in diabetic groups.

At the end of experiment, a markedly increase was observed in the heart wet weight of CIH diabetic rats versus normoxia diabetic rats. Since hypoxia treatment obviously translated to increased left ventricle to heart weight ratio it may be concluded that an anabolic pathways are triggered and can be distinguishable from diabetic pathologic hypertrophy of the heart and LV^35^ but it is different from hypoxia induced hypertrophy and have a more similarity to athlete’s heart that is a kind of normal and physiological adaptation of the heart to the stresses of aerobic exercise and physical conditioning.^36^

The characteristics of diabetic cardiomyopathy including disarray and collapse of myofibers and myocardial degeneration were found in LV sections of the diabetic groups with hematoxylin-eosin staining whereas the ventricular myocardium showed normal architecture under normoxia condition and rearrangement of diabetes induced abnormal architecture under normobaric hypoxia condition. Myocardial fibrosis showed approximately an eight fold increase in the diabetic rats, but CIH markedly diminished it to three fold. In disagreement with our finding, results of Lin et al in 2013 also showed that the intermittent hypobaric hypoxia exerted protective effects on rat hearts, by decrease in pro-apoptotic Bcl-2 family members, BNIP3 and Bad without any changes in architecture and fibrosis in short period of hypoxia.^10^ These results agreed with Lin et al.^10^

Lin et al findings suggest that intermittent hypobaric hypoxia exerts protective or deleterious effects on rat heart in a tightly time-course dependent manner.^10^ These data may partially explain controversial effects of acute hypoxia or ischemia on cardiac damage with CIH on cardiac protection.^10^

Altogether, it seems that chronic exposure to intermittent altitude is better stimulus for glucose disposal and improve symptom of cardiomyopathy. The present data showed that CIH have beneficial effects on heart of diabetic’s rat. Finally our study suggests that chronic intermittent altitude may be count as a potentially effective non-pharmacological strategy for the prevention and relief of cardiac symptoms of diabetic patients.

## Conclusion

Based on the presented results, CIH improves glucose homeostasis, regulates metabolic parameters, ameliorates the heart tissue fibrosis and finally switches the diabetic hypertrophy to physiological hypertrophy in heart.

Thus, we can suggest that chronic exposure to intermittent hypoxia (mimics of altitude) as a noninvasive intervention, may be a good choice to control of glucose and prevention of d diabetic cardiomyopathy.

## Acknowledgments

This work was supported by the Faculty of Physical Education and Sport Sciences of Tabriz University (Tabriz, Iran), special thanks goes to Dr. M,Farhoudi to permit us to perform our experiments in Neuroscience Research Center (NSRC) of Tabriz University of Medical Sciences(Tabriz, Iran).

## Competing Interests

Authors declare no conflict of interest in this study.

## Ethical issues

The compliance of all steps of this experiment with the National Institutes of Health publication (revised in 1996) were approved by the Animal Care and Use Committee of Tabriz University of Medical Sciences (Tabriz, Iran).
